# Ions at Helium Interfaces: A Review

**DOI:** 10.3390/e28010109

**Published:** 2026-01-16

**Authors:** Paul Leiderer

**Affiliations:** Physics Department, University of Konstanz, 78457 Konstanz, Germany; paul.leiderer@uni-konstanz.de

**Keywords:** helium interfaces, positive and negative ions, ion trapping, plasma resonances, 2D crystallization, electrohydrodynamic instability

## Abstract

Ions in liquid helium exist in their simplest form in two configurations, as negatively charged “electron bubbles” (electrons in a void of about 35 Å in diameter) and as positive “snowballs” (He^+^ ions surrounded by a sphere of solid helium, about 14 Å in diameter). Here, we give an overview of studies with these ions when they are trapped at interfaces between different helium phases, i.e., the “free” surface between liquid and vapor, but also the interfaces between liquid and solid helium at high pressure and between phase-separated ^3^He-^4^He mixtures below the tricritical point. Three cases are discussed: (i) if the energy barrier provided by the interface is of the order of the thermal energy k_B_*T*, the ions can pass from one phase to the other with characteristic trapping times at the interface, which are in qualitative agreement with the existing theories; (ii) if the energy barrier is sufficiently high, the ions are trapped at the interface for extended periods of time, forming 2D Coulomb systems with intriguing properties; and (iii) at high electric fields and high ion densities, an electrohydrodynamic instability takes place, which is a model for critical phenomena.

## 1. Introduction

Since the first liquefaction of helium gas, the condensed phases of helium have been studied intensely because of their unique properties and as model systems for condensed matter physics; the most well-known examples of these are probably the superfluidity of systems of bosons (^4^He) or fermions (^3^He), but also the quantum crystals ^3^He and ^4^He and the interfaces between the condensed phases of helium. In these investigations, ions in helium have often played a prominent role because they can be used as sensitive and convenient probes for studying helium properties. They are, however, also interesting objects by themselves, both as individual objects and—due to their Coulomb interaction—as systems displaying novel collective phenomena.

The ions mainly considered here are the two species—positively and negatively charged, respectively—that are most readily produced in liquid helium, e.g., by irradiation with high-energy particles, or by an injection of charge into the liquid with a field emission or a field ionization tip [1,2]. The negative ion is an electron trapped within a spherical void in the liquid, commonly referred to as an electron bubble. This structure is approximately 35 Å in diameter, maintained by a balance between inward-acting surface tension and the outward pressure from the electron’s zero-point motion. In contrast, positive helium ions are thought to resemble compact “snowballs” [3]. These form as a result of the electrostrictive forces generated by the ion’s central charge, leading to a solid-like configuration with a typical diameter of about 14 Å. Such ions have been used, e.g., for the imaging of quantized vortices in rotating superfluid ^4^He, as nucleation sites for cavitation and for studies of quantum turbulence [4,5,6,7]. Ions and their critical velocities for the generation of rotons and vortices in superfluid helium have been studied by McClintock and coworkers [8,9,10,11].

In this (partly historical) review, ions at *helium interfaces* are in the focus. In Section 2, we shall discuss the trapping of ions below the surface of liquid helium, at the interface of phase separated ^3^He-^4^He mixtures and at the interface between liquid and crystalline ^4^He. Electrons in surface states *above* the liquid–vapor interface of helium, which form similar two-dimensional Coulomb systems, have been treated in many reviews [12,13,14,15,16] and will not be considered here, except briefly in Section 4. Section 3 is devoted to the properties of these trapped ion entities in their interaction among themselves and with surface and bulk excitations of the helium. Finally, in Section 4, the electrohydrodynamic instability will be discussed: a phenomenon arising at high electric fields due to the electrostatic pressure exerted by the ions upon the interface, where they are trapped.

## 2. Trapping of Ions at Helium Interfaces

### 2.1. Ions at the Liquid–Vapor Helium Interface

It was already concluded in some of the first investigations of ions in liquid helium, from the quite different behavior of positive and negative ions, that the positive ion is a cluster of polarized atoms around one charge, whereas the negative ion is a large cage where the electron is self-trapping [3,17,18]. In a simple model, the radius, *R*, of the electron bubble can be obtained by minimizing the energy, *W*, of this complex

*W* = ℏ^2^π^2^/[2*mR*^2^(1 + ℏ/(*R*^2^2*mV*_0_)^1/2^)^2^] + 4π*R*^2^*σ* + (4π/3)*R*^3^*p* − e^2^(*ε* − 1)/2*εR* (1)

resulting from the energy of an electron in a spherical potential well of depth *V*_0_ and radius *R*, from the surface tension *σ*, the hydrostatic pressure *p* and the polarization of the surrounding medium with a dielectric constant *ε* [1]. The energy of a positive ion consists of the energy of the solid core and also the polarization of the liquid surrounding the snowball.

*W* = *W*_core_ − e^2^(*ε* − 1)/2*εR* (2)

where the energy *W*_core_ contains the energy of the snowball itself and the contribution of the liquid–solid surface tension.

A few years after these first ion experiments in helium, Bruschi et al. described a measurement in which the existence of a small potential barrier to the extraction of negative ions from the liquid into the vapor was demonstrated [19]. For positive ions, on the other hand, the potential barrier was so high that no appreciable current across the liquid–vapor interface could be observed.

**Figure 1:**
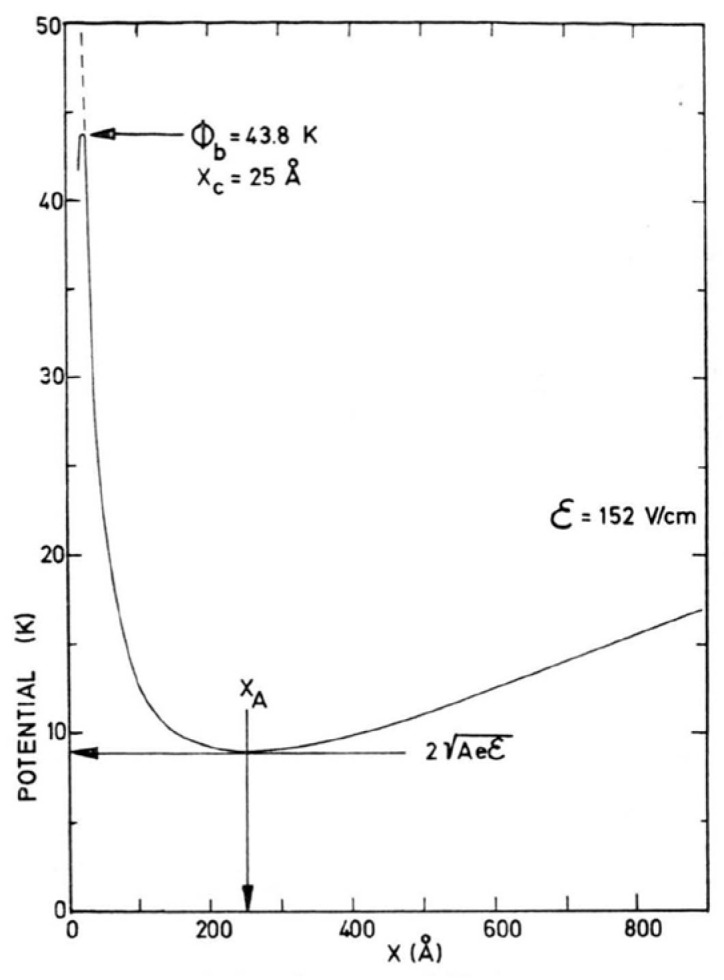
The image potential *V*(*x*) near the surface of liquid ^4^He as a function of distance *x* into the liquid. Reproduced with permission from [20].

The mechanism by which the negative ions cross the surface energy barrier was investigated by Schoepe and Rayfield [20,21]. The barrier is induced by the dielectric image potential, acting on the electron bubbles below the liquid–vapor interface (Figure 1). Originally, the authors interpreted their data in terms of thermal diffusion across the barrier [20]. This interpretation was revised in a second publication shortly afterwards [21], in which the escape of the electrons from their bubble state in the liquid through the free liquid surface into the vapor phase was ascribed to a tunneling process.

**Figure 2:**
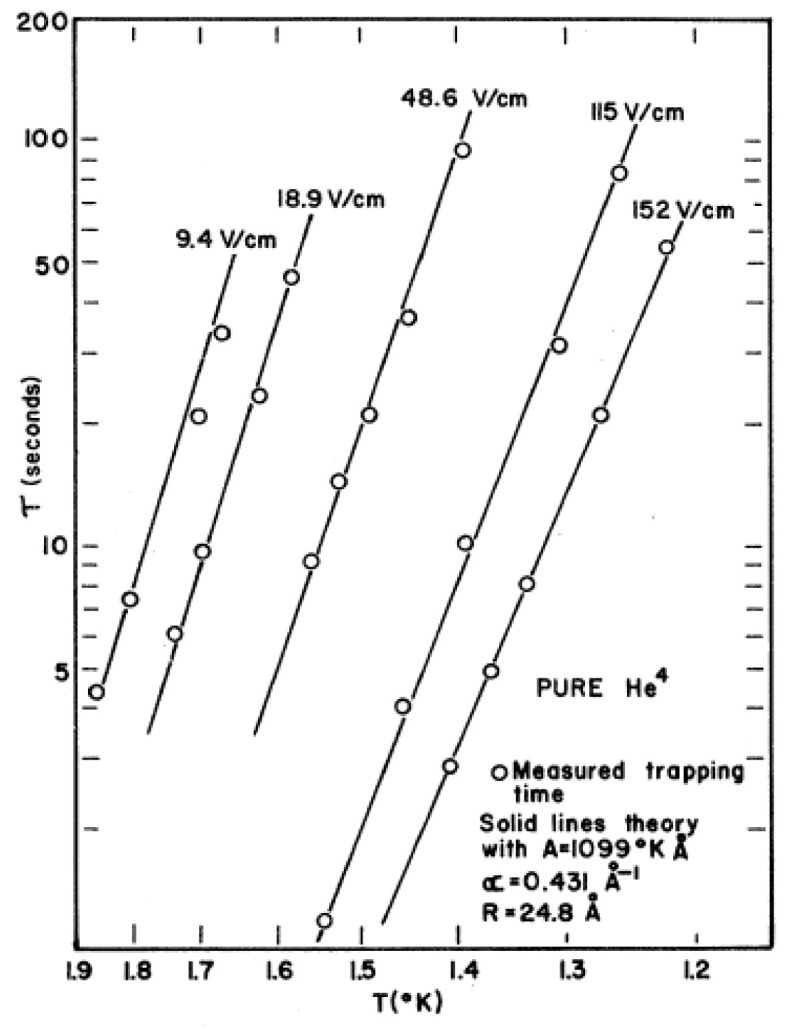
Experimental measured trapping times, *τ*, as a function of temperature, *T*, for pure ^4^He. *τ* varies from about 1 to 100 s. Reproduced with permission from [21].

The escape rates calculated in terms of this tunneling model were in good agreement with the experimental data shown in Figure 2, except that the absolute values of the calculated radii of the electron bubbles had to be assumed to be 26 Å and 32 Å for ^4^He and ^3^He, respectively: about 50% larger than derived from other experiments. When the accepted bubble radii from other sources were used instead, the calculations yielded trapping times that were 200 to 300 times longer than the experimentally observed values. This problem was further investigated by Cole and Klein [22], who instead of the semiclassical WKB method used in [21], applied the tunneling Hamiltonian method of Bardeen. Also, their results could be matched with the experimental data only when the radii of the bubbles were assumed as in Ref. [21]. Ancilotto and Tiogo [23], using a density functional method, reexamined this topic and, in particular, studied the behavior of the electron bubble as it approaches the free surface of liquid ^4^He. They found a value for the bubble radius in the bulk liquid of 17.9 Å, in line with the previous accepted results. Furthermore, it was found that the electron bubble is stable up to an electron-surface distance of *d*_0_ ≈ 23 Å; at smaller distances, it becomes unstable, bursts and releases the electron into the vapor phase. At distances, *d*, larger than *d*_0_, where the bubble is mechanically stable, the electron can still escape across the interface by quantum tunneling. The authors also examined both ingredients that are relevant here: namely, the tunneling probability per unit time and the bubble distribution function *n*(*x*), which gives the probability of a bubble being at a distance of *x* from the surface. At variance with previous theories, where a Boltzmann-like distribution function *n*_eq_ (*x*) was assumed, their finding is that *n*(*x*) deviates largely from this equilibrium form, *n*_eq_ (*x*), in the region close to the surface, where tunneling is most effective. Building on this result, they calculate trapping times, *τ*, which are now much smaller than in Refs [21,22] and in “semiquantitative” agreement with experiment. Yet, a discrepancy by a factor of 5 to 10 still persists [23].

Schoepe and Rayfield also report data for the trapping of negative ions in two ^3^He-^4^He mixtures, which are mostly in the range between the trapping times of the pure phases. As pointed out by the authors, some details of their results for the mixtures were peculiar and not understood [21]. To our knowledge, the trapping of electron bubbles at the free surface of He mixtures has not been studied further.

**Figure 3:**
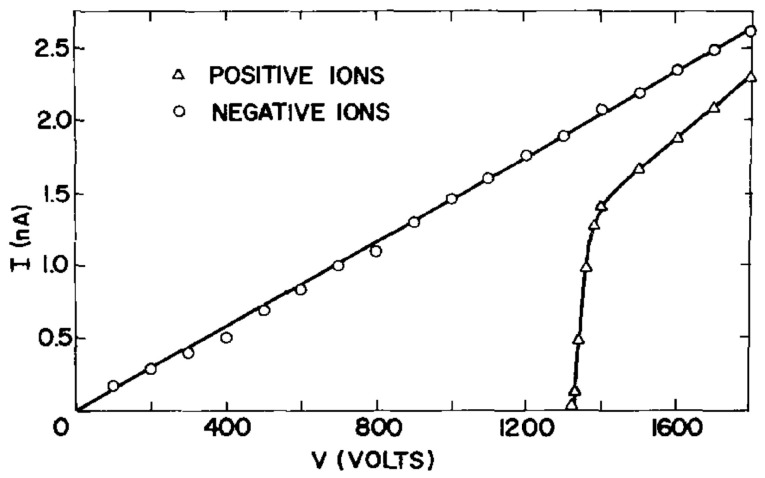
*I*-*V* characteristics for the transport of negative and positive ions across the liquid–vapor interface of ^4^He at a temperature of *T* = 4.2 K [24]. Reproduced with permission from [24].

For positive ions, Boyle and Dahm [24] found that—in line with previous results—under similar conditions, no current could be detected as long as the electric field was below some threshold value (see Figure 3). The current observed above this critical field can be ascribed to an electrohydrodynamic instability (Section 4).

### 2.2. Ions at the Liquid–Liquid Interface of Phase-Separated ^3^He-^4^He Mixtures

Liquid mixtures composed of ^3^He and ^4^He, near their consolute (tri-)critical point—at a temperature of *T*_t_ = 0.867 K and a ^3^He concentration of *x*_t_ = 0.67—serve as an exemplary system for studying higher order critical phenomena. These mixtures have been investigated with respect to their critical exponents: in particular, the interface separating the two coexisting phases, a heavier, superfluid ^4^He-rich and a lighter, normal ^3^He-rich phase [25,26]. This boundary exhibits pronounced softening as the system approaches the tricritical temperature, reflected in a sharp decline in the interfacial tension, which scales with the square of the temperature difference from the critical point, following the relation *σ* ∝ (*T*_t_ − *T*)^2^ [26].

**Figure 4:**
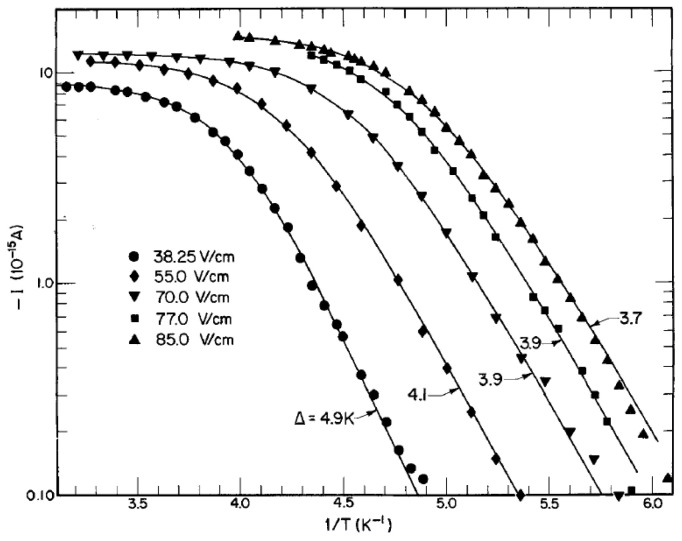
The observed temperature dependence of the negative-ion current crossing a ^3^He-^4^He phase boundary for several values of the applied electric field. The smooth curves show least-square fits to the experimental points using the phenomenological model of Kuchnir et al. [27] for the temperature dependence of the current. Reproduced with permission from [27].

For temperatures far below the tricitical point, the behavior of negative ions at the interface of such phase-separated ^3^He-^4^He mixtures has been studied by Kuchnir et al. [27]. They found a thermally activated ion current through the interface when the electron bubbles came from the ^4^He-rich side (Figure 4). The nearly exponential dependence at low temperatures suggests the presence of a potential barrier, which, depending on the applied electric field, is between 3.7 and 4.9 K high, and was ascribed to an electrostatic origin similar to the barrier for ions below the free liquid helium surface.

In the opposite direction, from the ^3^He-rich side, a negative ion current could not be observed, due to a high potential barrier with an estimated height of Δ*W* ≈ 220 K arising from the bubble’s self-energy [27]. Thus, electron bubbles can be trapped above the interface at a sufficiently low temperature. As the system approaches the tricritical point, the distinction between the two liquid phases diminishes, leading to a rapid reduction in the energy barrier. Once the barrier energy Δ*W* becomes comparable to the thermal energy k_B_*T*, ion transport across the interface becomes feasible via thermal activation, as is shown later (see below).

For positively charged helium “snowballs,” the interface acts as a potential barrier in the upward direction—from the denser to the less dense phase—due to the lower energy of positive ions in the superfluid phase, whose polarizability is higher [28,29]. When an appropriate electric field is applied, these ions can therefore accumulate just beneath the interface. As the system approaches the tricritical point, the energy barrier also diminishes in this case, allowing the ions to pass into the other phase once the barrier height becomes comparable to k_B_*T*.

**Figure 5:**
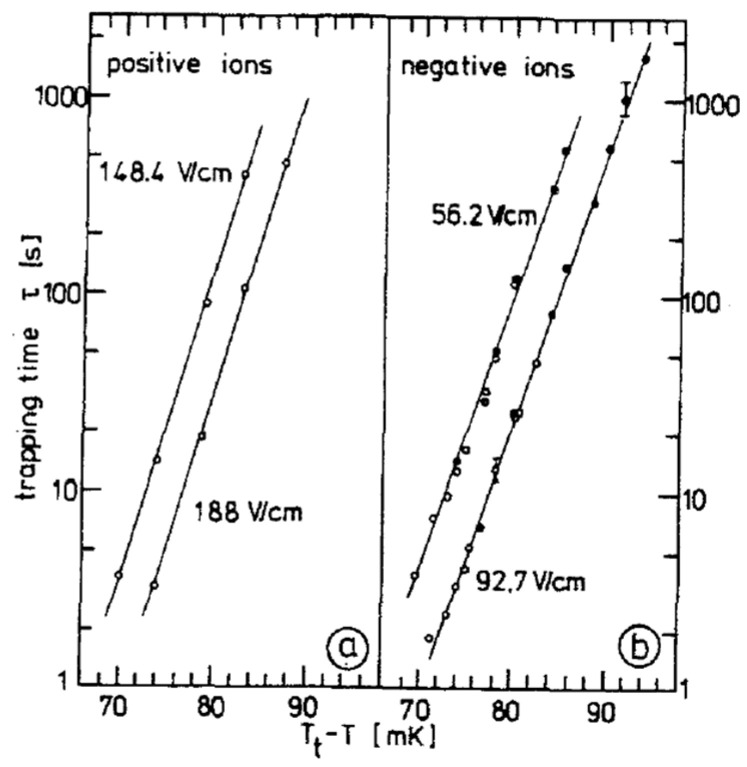
Trapping time, *τ*, of positive (**a**) and negative (**b**) ions at the interface of a phase-separated liquid ^3^He-^4^He mixture in the vicinity of the tricritical temperature, *T*_t_ = 0.867 K. The direction of the ion transport is from the lower to the upper phase for positive ions and in the opposite direction for negative ions. Electrical holding fields are indicated. Open and closed circles refer to electrical and optical measurements. Reproduced with permission from [28].

Because the barrier energy, Δ*W*, is connected to the structure of the ions, evaluating the trapping time, *τ*, at the interface—largely governed by Δ*W*—offers a sensitive consistency check for the theoretical models of ion structure and the influence of the interface. Measurements of *τ* have been made either by monitoring the decay of the electrical current following interface charging or by observing the optical distortion of the interface induced by the electrostatic pressure from the trapped ions [28,29], which was originally noticed by Crandall and Williams for electrons above the free surface of ^4^He [30] (see also Figure 15a in Section 4). Figure 5 presents the temperature-dependent behavior of the trapping time, *τ*, for positive and negative ions in the vicinity of the tricritical point.

The data reveal a steep temperature dependence, which, combined with the practical detection range of *τ* (approximately 1 to 1000 s using the previously described methods), confines the experimental observations to a narrow temperature window of 70 to 90 mK below the tricritical temperature *T*_t_. Beyond the temperature effects, *τ* also varies with the strength of the applied electric holding field, E; for both ion types, an increase in E leads to a reduction in *τ*, in agreement with theoretical expectations. Surprisingly, however, the magnitude and temperature dependence of *τ* are nearly identical for both positive and negative ions—an outcome that contrasts sharply with the predictions of the conventional snowball and bubble models, which anticipate distinct behaviors for each ion species [29]: (i) for negative ions, an evaluation of the bubble energy (Equation (1)) for the ^3^He- and the ^4^He-rich phase yields only a relatively small expected energy barrier on the order of 2 K at a temperature of *T*_t_ − *T* = 80 mK; (ii) for positive ions, on the other hand, the energy barrier Δ*W* calculated from Equation (2) has a value of 40 K at a temperature *T*_t_ − *T* = 80 mK, which is a factor of 20 higher than for negative ions. Considering the fact that the difference in the dielectric constant, *ε*, between the ^3^He- and the ^4^He-rich phase varies linearly with (*T*_t_ − *T*) [25] and assuming that the core energy of the snowball *W*_core_ does not change between the two coexisting phases, one expects a temperature dependence of the trapping time, *τ* [29]

*τ* ∝ exp[(*T*_t_ − *T*)/k_B_*T*] (3)

An exponential relationship of this form leads to the straight lines in Figure 5 and is obviously in agreement with the experimental data. Still, the observed trapping times of the positive ions are not fully explained by the model, because the calculated absolute value of Δ*W* is about a factor of two larger than that derived from the data in Figure 5a.

In contrast to positive ions, the calculated energy barrier, Δ*W*, for negative ions—based on Equation (1)—is significantly *smaller* than the experimentally observed values in Figure 5b, with the discrepancy nearly reaching an order of magnitude.

Given that the measurement uncertainty in the trapping times, *τ*, shown in Figure 5 is considerably smaller than these deviations, it is obvious that the simple ion models employed here are inadequate for a precise quantitative analysis. This suggests that more sophisticated theoretical treatments are required, for which the available data for *τ* could provide a sensitive consistency check.

### 2.3. Ions at the Liquid–Solid Interface of ^4^He

Experimentally, it has been shown that electron bubbles are not restricted to the liquid phase but are also present in solid helium [31]. By applying a calculation analogous to Equation (1), the energy of a bubble in solid ^4^He at melting pressure is estimated to be approximately 200 K higher than in the corresponding liquid phase [32]. This energy difference implies that it should be possible to confine electron bubbles at the boundary between liquid and solid helium. This trapping has been confirmed experimentally and has been utilized in various studies, such as determining the growth coefficient of ^4^He crystals [33]. In these measurements, the number of negative ions trapped at the interface did not change over many hours, indicating that the trapping time of negative ions is very long (see also Figure 22 in Section 4.3). For positive ion snowballs, on the other hand, it was observed that the liquid–solid interface could not be charged appreciably, because the trapping time of these ions at the interface was too short [32].

In summary for Section 2, the trapping of both ion species at the various helium interfaces can be qualitatively understood, but there are still deficiencies in the understanding of the details of the ion transport across these interfaces. Nevertheless, for low enough temperatures, the trapping times are long enough that ions can be collected at helium interfaces and kept there for a long enough time to form well-defined Coulomb systems with interesting properties, which will be discussed in the next chapter.

## 3. Ion Pools Below the Surface of Liquid Helium

As shown above, ions in liquid helium close to the free surface are trapped in a potential well, consisting of the repulsive image potential and the influence of the external electric field that pushes the charges towards the surface (Figure 1). Pointrenaud and Williams [34] have used these systems of trapped ions, both negative and positive, to obtain information about their properties in superfluid ^4^He. Using a resonance technique, they were able to measure the effective masses of both ion species. In their experiment, they applied an ac electric field perpendicular to the surface at a frequency *ω*/2π = 200 MHz, exciting vertical oscillations, and then determined the uniform external dc field at which the ions moved at resonance in their potential well. This allowed a direct calculation of the effective masses m* of the ions, which, at 0.7 K, were found to be *m*_+_* = 43.6 m_4_ and *m*_−_* = 243 m_4_, where m_4_ is the bare ^4^He mass. The ionic radii deduced from these masses were 6.0 Å and 17.9 Å for snowballs and electron bubbles, respectively [34].

**Figure 6:**
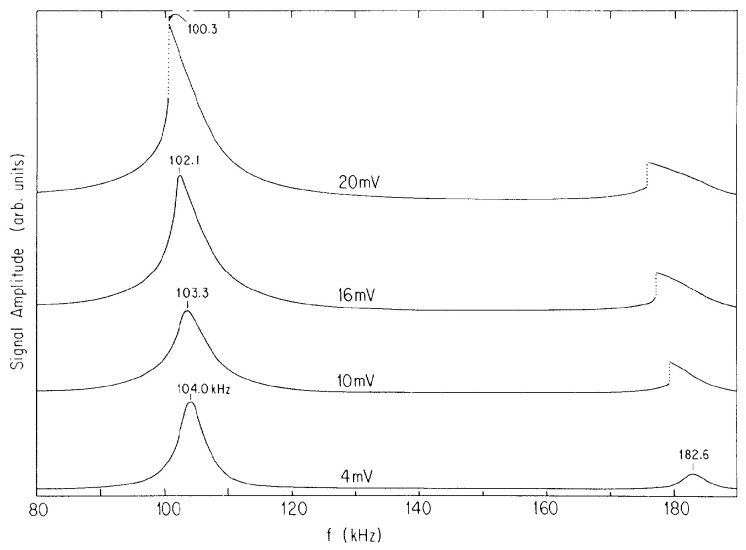
Plasma resonance spectra for positive ions at *T* = 0.22 K, displaying the resonance frequency shifts with increasing drive amplitude in millivolts, peak to peak. Reproduced with permission from [35].

Nearly a decade later, Ott-Rowland et al. [35] also applied a resonance technique to (in this case only positive) ions under the surface of liquid ^4^He, but now with the ac electric field applied parallel to the surface. In this way, plasmon resonances in the two-dimensional ion sheet could be excited, as shown in Figure 6. The ion effective mass derived from these resonances was found to be weakly dependent on temperature, and consistent with the value of *m*_+_* in Ref [34]. In addition, as also shown in Figure 6, strong nonlinear features were observed when the plasma resonances were driven at large amplitudes. This behavior was analyzed by Ikezi, who found that the ponderomotive force causes the nonlinear shift in the plasmon frequency [36].

In the following years, a series of experiments with ion pools underneath the surface of superfluid ^4^He were carried out in the group of Vinen at the University of Birmingham. Since a comprehensive review by Skrbek about this work has appeared recently [37], only some of the most relevant results for oscillatory modes of the ions in the pool and the Coulomb crystallization of the ions are summarized here.

**Figure 7:**
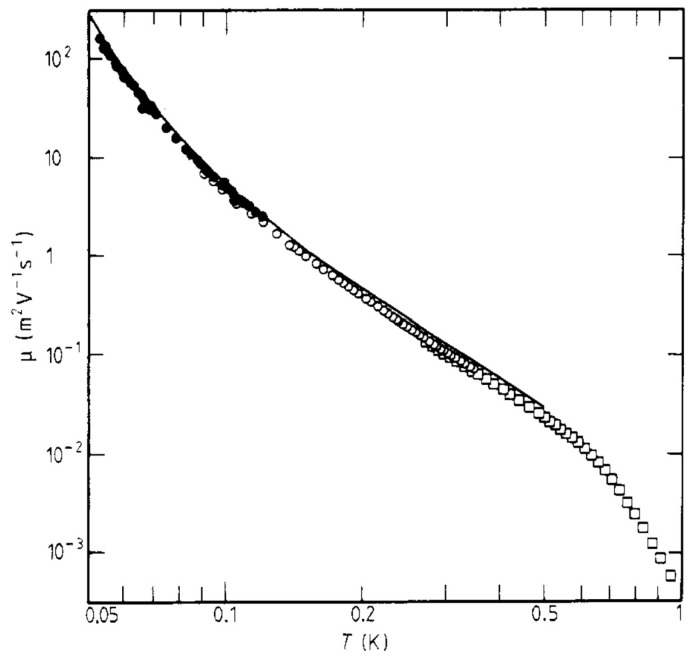
The mobility, *μ*, of negative ions trapped below the surface of ^4^He, plotted against temperature, *T*. Open circles, from the capacitance–conductance measurements and full circles, from the linewidths of the plasma resonances. The experimental results of Schwarz [39] are shown by open squares. (It should be noted that below about 300 mK, the high purity of ^4^He matters, otherwise the mobility displays a plateau due to scattering of the ions by ^3^He atoms. A technique for purifying ^4^He has been developed by McClintock [40].) Reproduced with permission from [38].

For the first time, Barenghi et al. [38] observed plasma resonances of negative ions. For this purpose, the system had to be cooled below 130 mK for the resonances to have a high enough Q (limited by the ion mobility). Both the effective mass and the mobility of the ions were determined from the frequencies and the widths of the resonances, respectively. For the negative ion mass, a value of (237 ± 7) m_4_ was found, in agreement with the result by Pointrenaud and Williams [34]. In the same work, the ion mobility was measured with a capacitance–conductance technique in the temperature range between 0.1 and 1 K, and as Figure 7 shows, the data for the ions close to the surface are in perfect agreement with the data of Schwarz for ions in the bulk liquid [39].

**Figure 8:**
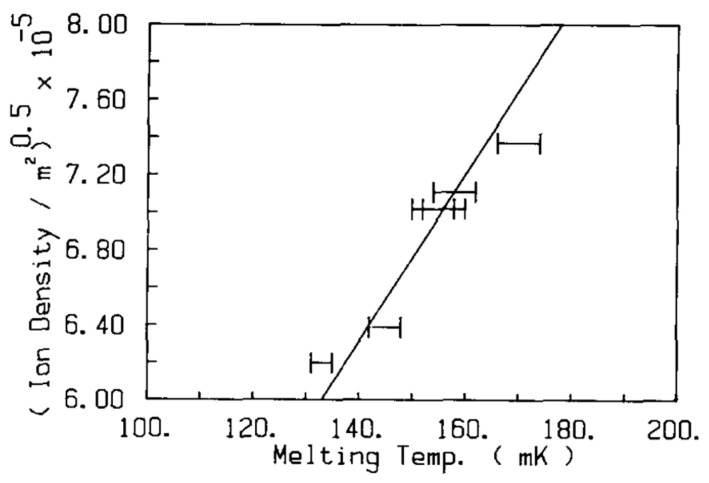
Melting curve of Coulomb crystals of negative ions, trapped at the liquid ^4^He surface, for ion densities between 3.8 and 5.5 × 10^11^ m^−2^. The solid line is for a plasma parameter of *Γ* = 130. Reproduced with permission from [43].

In the temperature range of the experiments in Ref. [38] (from 50 to 150 mK) and at the ion densities studied there, one would have expected that upon cooling, the ions in the pool undergo a phase transition from a classical 2D fluid to a 2D crystal, when the ratio between Coulomb energy and thermal energy, denoted by the plasma parameter *Γ*, reaches a critical value. A similar crystallization had been observed in 1978 by Grimes and Adams [41] in a system of free electrons above the surface of ^4^He for *Γ*∼130. However, in the experiment by Barenghi et al., no indication of Coulomb crystallization of the ions could be observed. It took four more years until Mellor and Vinen, using a sophisticated technique in which the so-called Shikin mode [42] of the ion-capillary wave system was excited, were able to prove the existence of Coulomb crystals and their melting at the expected temperatures [43,44] (Figure 8).

**Figure 9:**
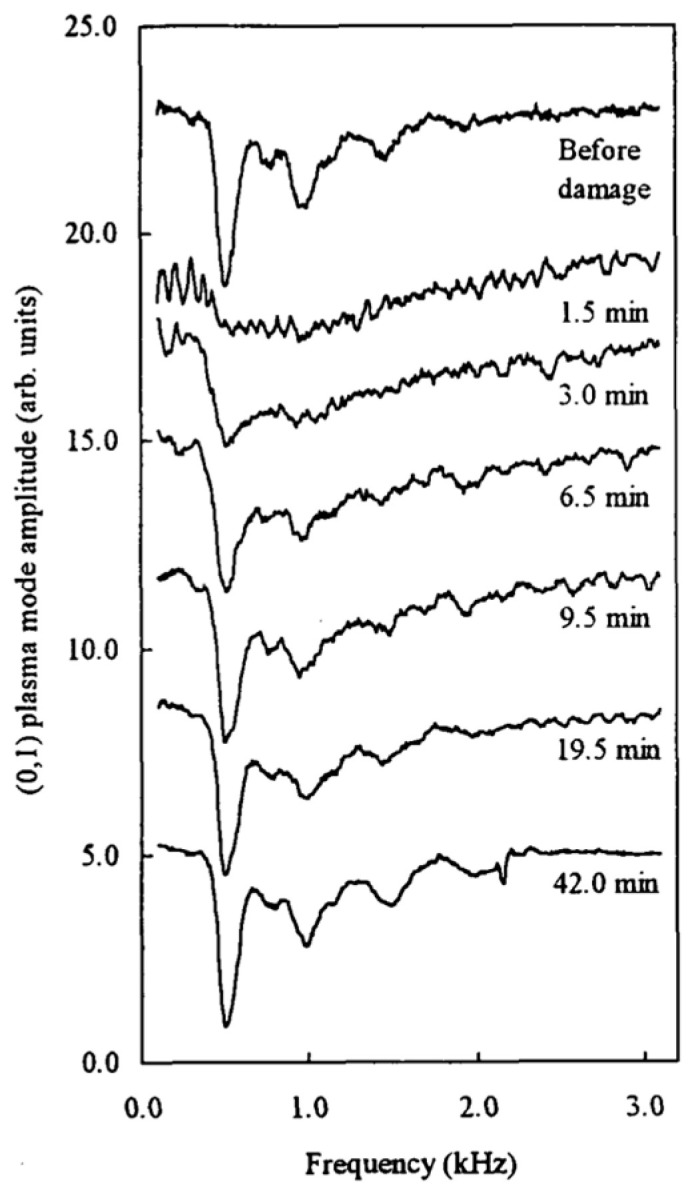
The time recovery of the spectrum of shear modes after damaging a 2D ion crystal by applying a high shear drive. The temperature was 17 mK, the applied magnetic field was 1.35 T, the charge density of the ion pool was 2.9 × 10^11^ m^−2^, the radius was 12.1 mm and the melting temperature was 125 mK. The ions were trapped 53 nm below the helium surface. Reproduced with permission from [45].

From the observation of both the fundamental and higher modes, it could be shown that these crystals have triangular symmetry, as in the case of electrons above helium [41]. Moreover, it was demonstrated that a carefully produced crystal can be damaged by applying a high electrical shear drive and, subsequently, the damaged crystal gradually recovers, similarly to the annealing in many 3D conventional solids [45] (Figure 9). In one of the following experiments on the transverse response of the ion pool [46], the authors even report evidence for the existence of a hexatic phase, which is predicted to exist between the 2D solid and the isotropic 2D liquid [47,48].

**Figure 10:**
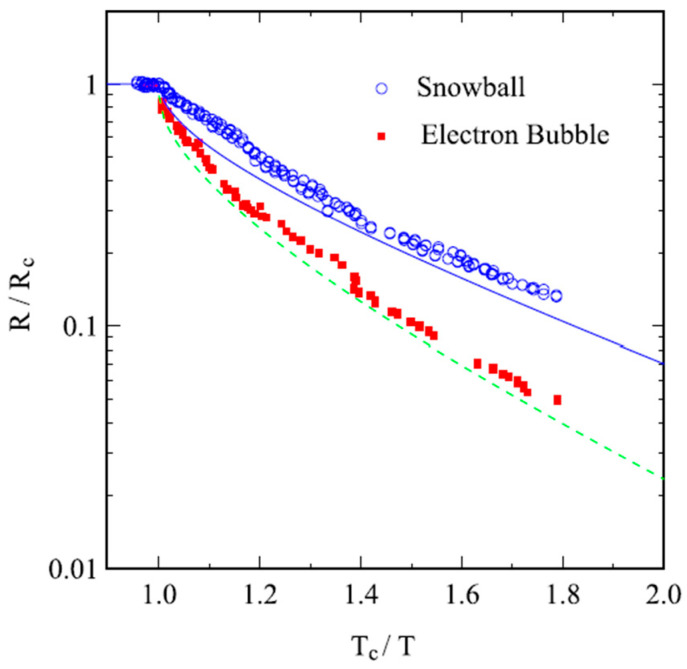
Normalized resistivity of electron bubbles and snowballs trapped below the free surface of superfluid ^3^He-B. Open circles are for snowballs and solid squares for electron bubbles. The solid line is the resistivity of the Wigner solid of electrons above the free surface. The dashed line is for the electron bubble mobility in the bulk, according to Kokko et al. [49], data points are from Shiino et al. [50]. *R*_c_ is the resistivity in normal ^3^He above *T*_c_. Reproduced with permission from [16].

Pioneering results with ions below the surface of liquid helium have also been obtained by Kono, Ikegama and coworkers [16]. In their work, mobility measurements of both positive and negative ions were used as a sensitive probe for the properties of the ^3^He superfluid phases ^3^He-A and ^3^He-B. The electrical resistivity of ions trapped below the free surface of ^3^He-B is shown in Figure 10. Its temperature dependence is discussed in terms of the scattering of quasiparticles in the superfluid; it drops rapidly as the temperature is lowered below T_c_, the transition temperature to the superfluid state, due to the decreasing number of quasiparticles.

In a continuation of this work, Ikegami et al. could demonstrate the existence of surface Andreev bound states at the ^3^He-B surface by an analysis of the temperature and depth dependence of the measured ion mobility, down to 250 µK [51]. These states form as a result of the nontrivial topology of ^3^He-B. For a review on this topic, see Ref. [52]. Another novel example for the use of ions to investigate superfluid ^3^He properties is the direct observation of spontaneous symmetry breaking leading to orbital chirality in superfluid ^3^He-A [53].

So far, properties of individual ions (mass, size, mobility), collective phenomena in ion pools (plasma excitations, Coulomb crystallization) and ions as a tool to study the liquid helium surface have been discussed. In the next chapter, effects at high electric fields and high charge densities will be reviewed, when the interface can no longer be considered as being undisturbed by the charges.

## 4. The Electro-Hydrodynamic Instability of Charged Helium Interfaces

**Figure 11:**
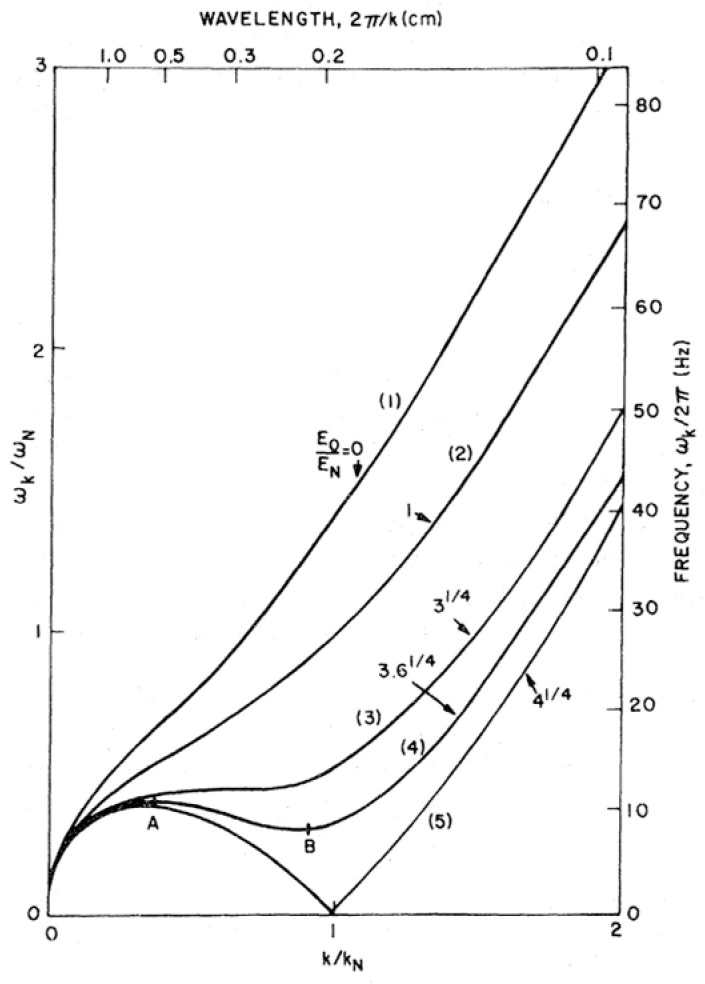
Calculated dispersion relation (angular frequency *ω* vs. wave vector *k*, in reduced units) of surface waves on a charged ^4^He surface at different values of the applied electric field (curves (1) to (5)). At the points A and B, the group velocity of the surface waves is zero. Reproduced with permission from [59].

Under the influence of the external electric holding field, trapped ions exert an electrostatic pressure upon the interface where they are collected. This leads to a slight depression or rise in the charged interface, depending on whether the ions are trapped above or below the interface, respectively. As the electric field is raised, this deformation increases in amplitude, and it is expected that the interface will undergo an instability with a breakthrough of the charges. This electro-hydrodynamic (EHD) instability, first considered by Frenkel [54] and Tonks [55] for classical charged liquid surfaces, was studied theoretically by Gorkov and Chernikova for the case of surface-state electrons above liquid helium [56]. From their analysis, they concluded that the flat surface of liquid helium can only be charged up to a maximum charge density of ∼2 × 10^9^ electrons/cm^2^. This was experimentally confirmed by Rybalko and Kovdrya [57]. Volodin et al. [58] investigated the conditions for the appearance of the instability in more detail, as well as the mechanism whereby the electrons leave the helium surface. They observed that when the instability sets in and surface waves build up, sharp dips develop at the surface, from which charged bubbles split off and move to the anode plate immersed in the liquid, leaving the surface in chaotic motion. Gorkov and Chernikova, however, had predicted that a periodic superstructure might appear on the charged surface, with dimensions on the order of the capillary length *a* of liquid helium given by *a* = (*σ*/Δ*ρg*)^1/2^ (where *σ* is the surface tension, Δ*ρ* is the density difference between the lower and the upper phase and *g* is the acceleration due to gravity). For ^4^He at 1 K, the value of *a* is about 0.5 mm. As a precursor to the instability, softening of surface waves should occur (see Figure 11). This was further investigated theoretically by Mima and Ikezi, again for the example of surface-state electrons on liquid helium [59].

The first experimental verification of this softening, however, was not for the system of surface-state electrons on liquid helium, but for ions at the interface of phase-separated ^3^He-^4^He mixtures, as shown in the following section.

### 4.1. Ion-Induced Ripplon Softening

As already mentioned in Section 2, the interface of phase-separated ^3^He-^4^He mixtures presents a potential barrier for both positive and negative ions and allows for trapping of these ions over long periods of time, provided that the temperature is not too close to the tricritical point. Negative ions can be collected at the interface from above, and positive ions from below.

*Without* the presence of charges, the dispersion relation of surface waves (ripplons) at the ^3^He-^4^He interface is given by [27]

(*ρ*_4_ + *ρ*_3_)ω^2^ = (*ρ*_4_ − *ρ*_3_)*gq* + *σq*^3^ (4)

The contributions on the right-hand side originate from gravity and the interfacial tension, denoted by *σ*. *ρ*_3_ and *ρ*_4_, represents the densities of the ^3^He- and ^4^He-rich phases and *g* is the gravitational acceleration. The damping of the ripplons is neglected here. When ions are added, Equation (4) has to be adapted because any local distortion of the interface caused by ripplons redistributes the initially uniform ion population. This redistribution generates a spatially varying, wave-vector-dependent ion pressure. As a result, the restoring forces in Equation (4) are reduced, which lowers the ripplon frequency and introduces an additional term in the dispersion relation [56]:

(*ρ*_4_ + *ρ*_3_)*ω*^2^ = (*ρ*_4_ − *ρ*_3_)*gq* + *σq*^3^ − *E*^2^*q*^2^/4π (5)

where it is assumed that the interface is charged to saturation (i.e., the electrical field *E* is only nonzero in the half space between the ion pool and its counter electrode; the ion density is then *n* = *E*/4πe). Equation (5) implies the softening of ripplons, which is shown in Figure 11 and is most pronounced for the wave vector *q*_c_ =1/*a* (In Figure 11, *k*_N_ corresponds to *q*_c_). At the critical field *E*_c_ = [64π^2^(*ρ*_4_ − *ρ*_3_)*gσ*]^1/4^, the frequency of the mode with *q* = *q*_c_ reaches zero. For *E* > *E*_c_, *ω* becomes imaginary, which implies that the interface becomes unstable against deformations with wave vectors ≈ *q*_c_.

**Figure 12:**
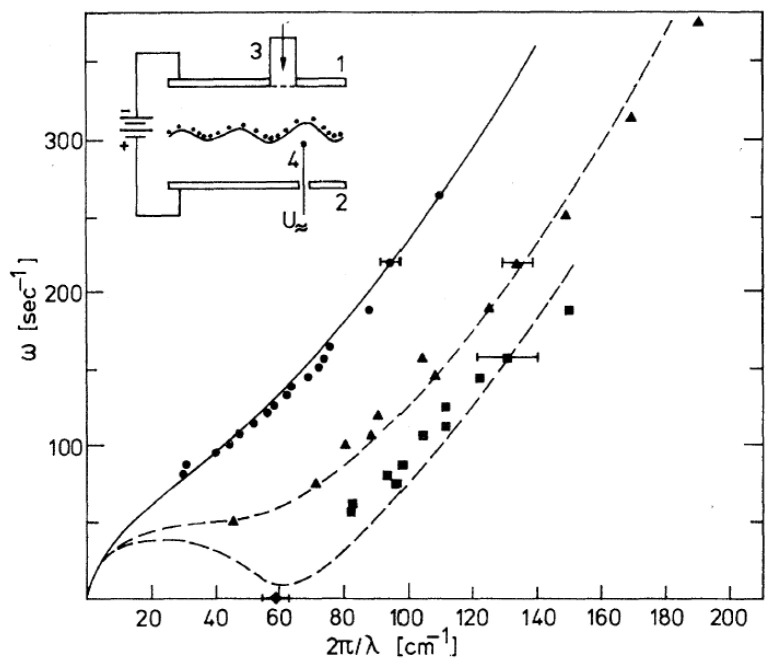
Dispersion relation of ripplons at the interface of a phase-separated ^3^He-^4^He mixture, completely charged with negative ions from above, at *T* = 0.567 K. The angular frequency *ω* vs. the wave vector *q* = 2π/*λ* is plotted. Full circles, *E* = 111 V/cm; triangles, 1000 V/cm; squares, 1119 V/cm; and diamonds, 1145 V/cm. The dashed curves are calculated according to Equation (5). The dispersion relation for the uncharged interface is given by the solid line (Equation (4)). The inset shows the schematic set-up: 1, 2—top and bottom capacitor plates; 3—field emission tip; and 4—wave generator. The wave amplitude is largely exaggerated. Reproduced with permission from [60].

Experimentally, the dispersion of ripplons at the interface of phase-separated ^3^He-^4^He mixtures was determined in Ref. [60] with a setup that is schematically shown in the inset of Figure 12. The interface, positioned between two capacitor plates, was charged from above by using negative ions emitted by a field-emission tip. The resulting distortion of the surface, illustrated in Figure 15a, resembles the effect that was previously observed for electrons at a free surface by Williams and Crandall [30]. To excite interfacial waves, a thin horizontal wire was placed just beneath the interface and driven with an alternating voltage of angular frequency, *ω*. The ripplon wavelength was then measured by scanning the wave pattern with a narrow optical beam [26].

Ripplon dispersion curves obtained in this way for the negatively charged interface are shown in Figure 12 for three different electric fields at a temperature of 0.567 K. At the lowest field, the data points nearly coincide with the dispersion curve for the uncharged interface, but as the electric field is increased, the softening of the ripplons becomes quite obvious.

**Figure 13:**
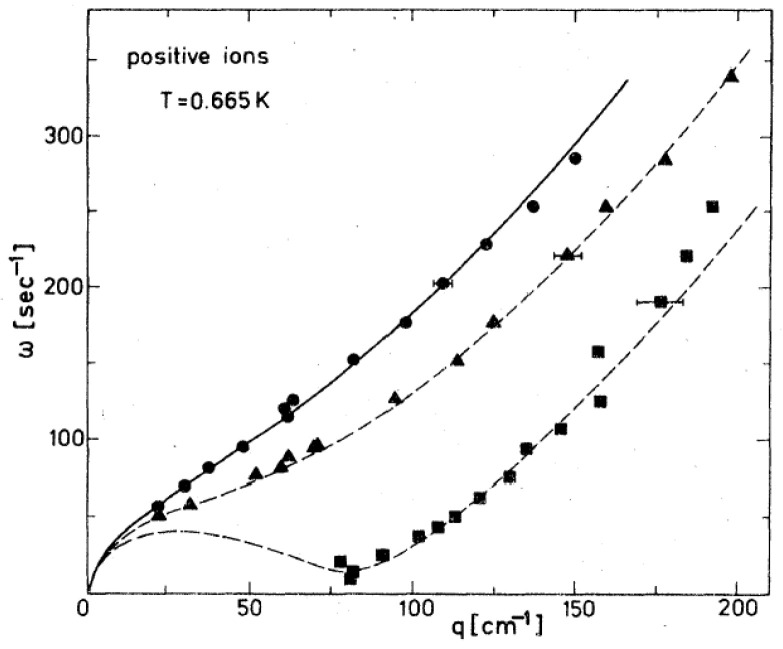
Dispersion relation of ripplons at the interface of a phase-separated ^3^He-^4^He mixture, completely charged with positive ions from below, at *T* = 0.665 K. The interface becomes unstable at *E*_c_ = 875 V/cm, corresponding to an ion density of *n*_c_ = 4.8 × 10^8^ cm^−2^. Full circles, *E*/*E*_c_ = 0.12; triangles, 0.71; and squares, 0.995. The dashed curves are calculated according to Equation (5). The dispersion of the uncharged interface is given by the solid line (Equation (4)). Reproduced with permission from [61].

Figure 13 shows data at a somewhat higher temperature: 0.665 K [61]. In this case, the interface was charged with positive ions from below, but the ripplon softening was similar, as expected. Since the temperature was now closer to the tricritical point and the interfacial tension was therefore smaller [26], the capillary length decreased and the critical wave vector shifted from *q*_c_ = 63 cm^−1^ to *q*_c_ = 82 cm^−1^.

Of the ripplons investigated, those with the critical wave vector *q*_c_ = 1/*a* are of particular interest, since they should finally lead to the instability of the interface at *E* = *E*_c_. As the electric field is increased, the angular frequency of ripplons with wave vector q_c_ should be reduced, according to Equation (5), to

**Figure 14:**
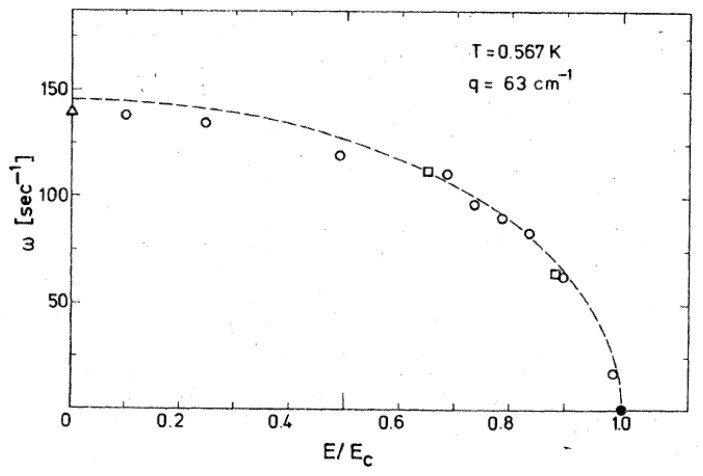
Frequency of interfacial waves in ^3^He-^4^He at a temperature of *T* = 0.567 K and a wave vector of *q*_c_ = 1/*a* = 63 cm^−1^. The interface is always charged to saturation. Circles represent measurements with positive and squares represent those with negative ions. The triangle refers to the uncharged interface. The dashed line shows the result of Equation (6). Reproduced with permission from [61].

*ω* = *ω*_0_ [1 − (*E*/*E*_c_)^2^]^1/2^ (6)

where *ω*_0_ is the frequency of ripplons at the uncharged interface. As Figure 14 shows, the data for both negative and positive ions are in good agreement with this expectation.

**Figure 15:**
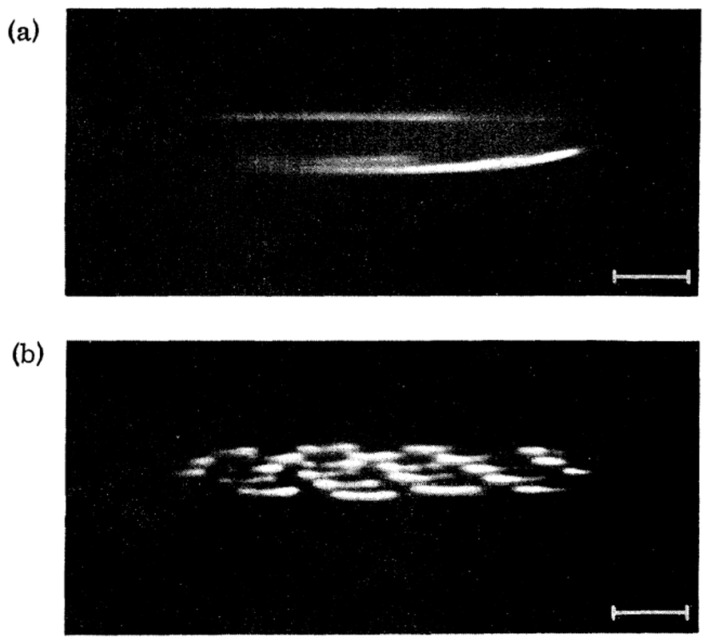
(**a**) Deformation of the ^3^He-^4^He mixture interface at *T* = 0.567 K and *E* = 1120 V/cm, observed from below at an angle of 6°. The elliptical bright lines are reflections of the nearly circular rim of the flat depression. The scale bar represents 1 mm. (**b**) The electrical field is increased to *E* = 1160 V/cm. The large depression has spontaneously deformed into an array of smaller dimples. Reproduced with permission from [60].

The softening of interfacial waves is reminiscent of structural phase transitions in solid-state physics. In crystals, a soft mode is often observed when the system approaches an instability point. Then, at the transition, a phase with a new symmetry appears. For charged helium surfaces, this new symmetry corresponds to the periodic superstructure predicted by Gorkov and Chernikova [56]. Such a structure was observed [60] when the applied electric field was increased beyond the critical value of *E*_c_ (provided that the charge density *n* was distinctly smaller than the value *n*_c_ of the fully charged surface at the critical electrical field *E*_c_, see below). The morphology of the depression was then markedly different: instead of a flat, homogeneous region roughly 1 cm wide, as shown in Figure 15a, the surface now exhibited a regular pattern of charged dimples spaced approximately 1 mm apart (Figure 15b).

**Figure 16:**
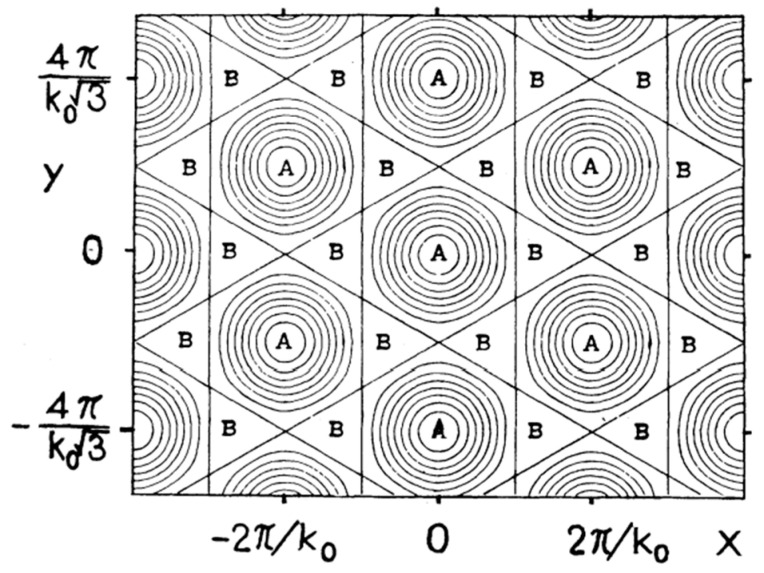
Calculated equal-height contours of the surface when the lattice is formed. A and B indicate the locations of the bottoms and the peaks, respectively. The contours equally divide the height. Reproduced with permission from [62].

A careful variation in the electric field around the value of *E*_c_ revealed that for *E* slightly above *E*_c_, first, a corrugated, bandlike structure developed—clearly linked to the soft ripplon mode, as evidenced by its identical wave length. During its formation, this band structure fragmented into discrete dimples, which arranged themselves into the regular pattern shown in Figure 15b. This phenomenon was reversible: by adjusting the external electric field, one could repeatedly go back and forth between a uniform and a periodically modulated charge distribution, without ion breakthrough occurring. For high charge densities, on the order of *n*_c_, charge breakthrough developed in a way much like the chaotic motion described by Volodin and Khaikin [58]. The symmetry of this “dimple crystal” was hexagonal, which was consistent with the prediction by Gorkov. This was further supported theoretically by Ikezi in Ref. [62], which appeared shortly after Ref. [60] (see Figure 16).

In addition to the periodic array in Figure 15b, it was observed in Ref. [60] that such charged dimples can also persist as individual structures. Each dimple contains approximately 10^6^ ions, so that dimples interact via strong Coulomb repulsion. The lattice of dimples is hence a macroscopic analog of a 2D Coulomb crystal [41,43].

Further investigations of the phenomenon of structure formation subsequent to an EHD instability were not carried out with the ions considered so far at the ^3^He-^4^He interface, but rather with electrons at the surface of liquid ^4^He. Although the present review is focused on *ions* at helium interfaces, a short overview of these studies with electrons will be given in the next section, because analogous effects are also expected for ions.

### 4.2. The Dimple Lattice

It was only a small step from the first observation of the dimple lattice at the ^3^He-^4^He interface to the experimental realization of this phenomenon with surface-state electrons on the free surface of liquid ^4^He [63]. Also, in this case, a hexagonal lattice of dimples was observed to form, yet under somewhat different conditions: since the surface tension and the density difference between the lower (liquid) and the upper (vapor) phase are larger than for the ^3^He-^4^He interface, the absolute value of the critical electric field is more than a factor of two higher; moreover, the lattice constant of the dimple crystal and the number of electrons per dimple are larger because of the larger capillary length of the liquid ^4^He surface.

As for the ^3^He-^4^He interface, two regimes were found for the development of the instability, depending on whether the charge density, *n*, is above or below a threshold density, *n*_t_ [64]: (i) for *n* > *n*_t_, as the electric field is slightly raised above *E*_c_ and then held fixed, some random surface indentation grows and charges break through (as already observed by Volodin and Khaikin [58]); (ii) for *n* < *n*_t_, the dimples which form at *E*_c_ grow only to a finite depth, and then arrange themselves in a stable configuration.

**Figure 17:**
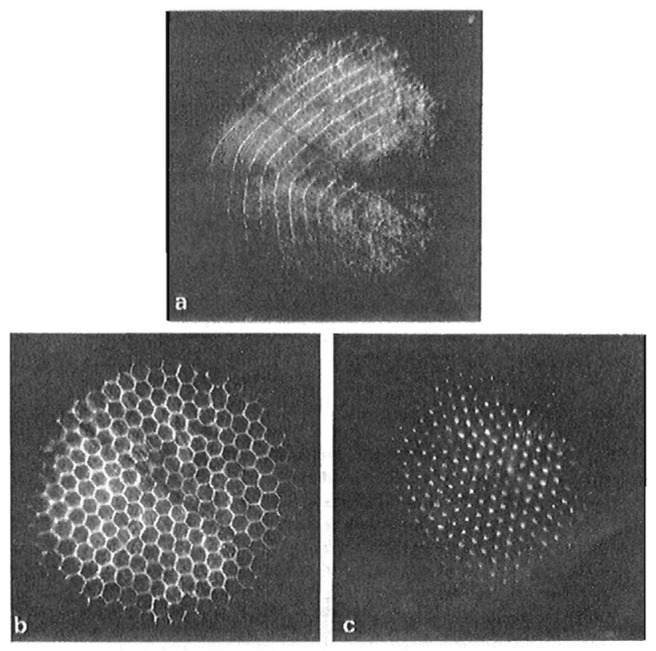
Formation of a dimple lattice on a liquid ^4^He surface (*T* = 3.5 K), charged with electrons from above. The picture shows the surface deformation approximately 2 s (**a**) and 6 s (**b**,**c**) after the field had been increased to *E*_c_. The image plane in (**a**,**b**) was chosen such that convex deformations of the surface, corresponding to local maxima, appear bright; in (**c**), bright areas correspond to local minima (i.e., the center of the dimples). The distance between adjacent rows of dimples is close to the wavelength 2π*a* of the soft ripplon: 0.24 cm, in this case. Reproduced with permission from [65].

An example for this latter case is shown in Figure 17. First, a wavelike structure appears, then troughs begin to split into dimples, until finally the whole charged surface is filled with this dimple lattice [65]. The structure formed in this way is stable; small perturbations like a slight vibration of the surface do not destroy it.

According to Ref. [64], the distinct nature of the two instability regimes arises from the relative strength of the electrostatic force acting on the surface. When the charge density, *n*, remains below the threshold, *n*_t_, the surface deformation stabilizes at a finite dimple depth due to the counteracting forces from gravity and surface tension. In contrast, once *n* exceeds *n*_t_, the electrostatic force is always dominant, overriding these stabilizing effects. As reported in Ref. [64], stable dimple lattices were observed up to a charge density of *n*_t_/*n*_c_ = 0.08 ± 0.03: a value of the same order of magnitude as the upper limit of 0.2 calculated by Ikezi [62].

Close to the instability, Ikezi predicted small hysteresis effects, which implies that in the language of critical phenomena, the transition is of (weakly) the first order. A small hysteresis was also observed experimentally [64,65]: once the dimple structure had developed at the critical field *E*_c_, *E* could be reduced by Δ*E* ∼ 5 × 10^−3^ *E*_c_ before the dimples disappeared and the homogeneous charge distribution was reestablished. At the transition, the depth of the dimples changed from *d* = 0 to *d*∼0.08 mm for the fully developed lattice.

**Figure 18:**
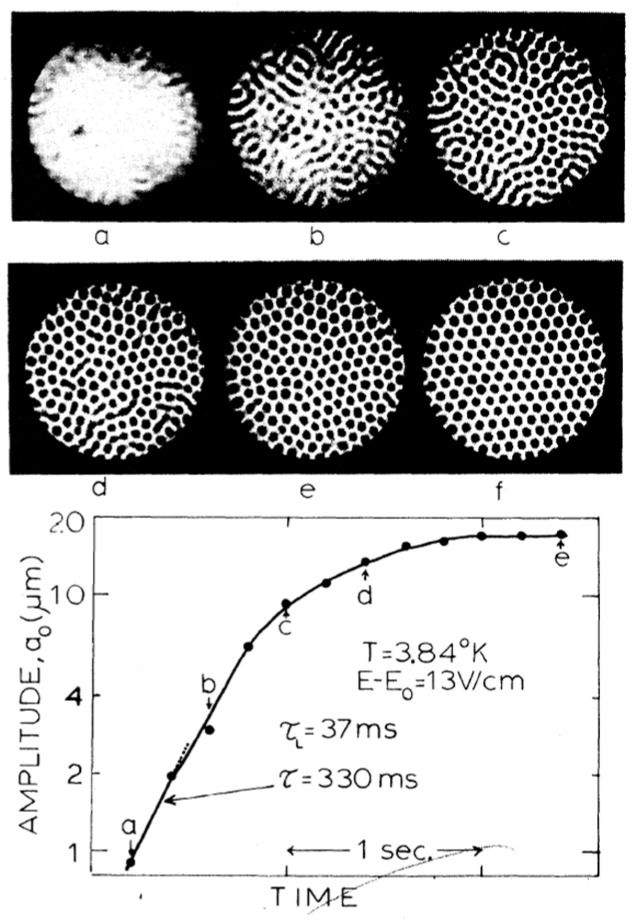
Time development of the surface deformation after a sudden field change. A glassy structure appears (**a**), but a perfect crystal (**f**) can eventually be formed with slow cycling of the electric field. (**b**–**e**) are intermediate states. *τ*_L_ is the linear growth rate, see Ref. [66]. Reproduced with permission from [66].

A more detailed study of the development of the dimple crystal from the homogeneous charge distribution and of hysteresis effects has been presented by Giannetta and Ikezi [66]. They report measurements of the nonlinear behavior of the surface near this instability point and compare their data with the existing theories on nonlinear equilibrium. In particular, they observe a precursor stripe phase with pronounced hysteresis, which is unexpectedly only present at relatively high temperatures around 4 K. From their study of hysteresis effects, they conclude that there are no electrons between the dimples, i.e., once the dimples are formed, their charge is fixed, which confirms the observation for the dimples at the ^3^He-^4^He interface [60]. In Ref. [66] also the character of imperfect dimple crystals is discussed: grain boundaries, dimple “glasses” and the growth time for these structures. As an example, Figure 18 shows the appearance of a glassy structure, which can gradually be transformed to a perfect crystal by careful “annealing,” using slow cycling of the electric field (cf. the annealing of an ionic crystal in Figure 9).

**Figure 19:**
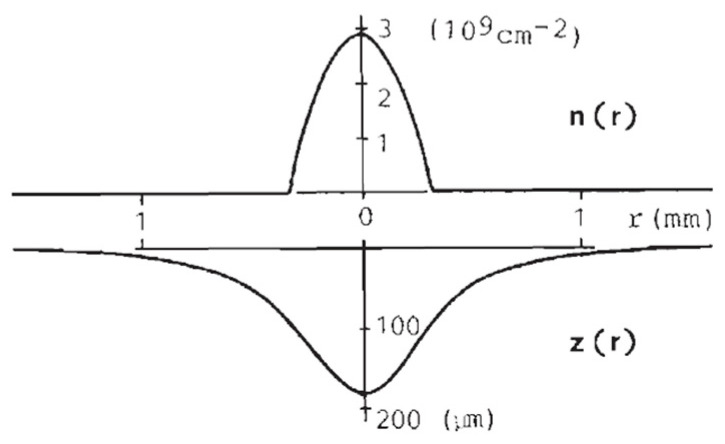
Calculated electron density *n*(*r*) and surface profile *z*(*r*) of a charged dimple on liquid ^4^He containing 5 × 10^6^ electrons in an electric field *E* = 3400 V/cm. The helium temperature is 2.5 K and the corresponding critical field *E*_c_ = (16π^2^*gσρ*)^1/4^ is 2600 V/cm [63]. Reproduced with permission from [67].

While Giannetta and Ikezi mainly considered dimple *arrays* and their formation, at the same time, Leiderer et al. studied the properties of *individual* dimples. In Ref. [67], the surface profile and the related charge distribution in the dimple were determined by a self-consistent numerical calculation, as shown in Figure 19.

**Figure 20:**
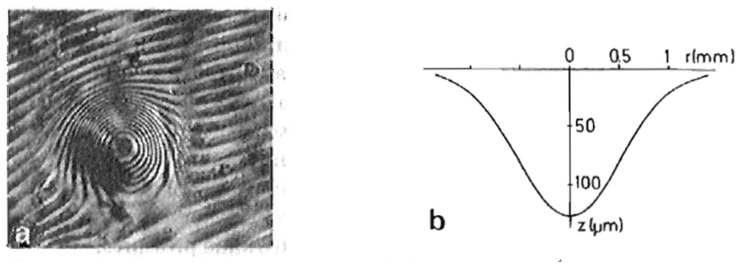
(**a**) Interference pattern of a macroscopic dimple on the surface of liquid ^4^He (*n*∼5 × 10^6^ electrons, *T* = 2.5 K, *E* = 2.9 × 10^3^ V/cm). The fringe pattern is asymmetric because the two interferometer plates were slightly tilted. Consecutive fringes correspond to a difference in surface height of 10.9 µm. (**b**) The surface profile derived from (**a**). Reproduced with permission from [65].

The result demonstrates that the charge of a dimple is concentrated close to the dimple center, so that the area between dimples is indeed free of charge (cf. Ref. [66]). Consequently, multielectron dimples can be considered as objects with a fixed charge, interacting via Coulomb repulsion, as also conjectured by Giannetta and Ikezi. This calculation was supported by an experiment where the profile of individual dimples was determined by an interferometry experiment (Figure 20) [65].

In his first theory for the dimple lattice, Ikezi assumed that the surface is an equipotential, i.e., that the electron density is nonzero everywhere and is only modulated along the surface [62]. As shown above [66,67], however, in the dimple state, all the electrons are deeply trapped in well-separated, non-sinusoidal dimples. Ikezi et al. [68] and Melnikov and Meshkov [69] have presented more extensive theories since then, where this is taken into account.

### 4.3. Beyond the Instability Point

Electrohydrodynamic instability can be considered as an analogy to a phase transition with a characteristic soft mode which develops as one approaches a critical point, e.g., of a binary liquid mixture, where spinodal decomposition sets in [70]. What happens when a system is quenched into the unstable regime beyond this instability point? From spinodal decomposition, it is known that in this unstable regime, not only one particular wave vector—the soft mode—grows as a function of time, but a whole band of wave vectors becomes unstable. This feature is also contained in the dispersion relation (Equation (5)), which governs charged helium surfaces or interfaces. Thus, these systems could serve as models for the general development of instabilities.

**Figure 21:**
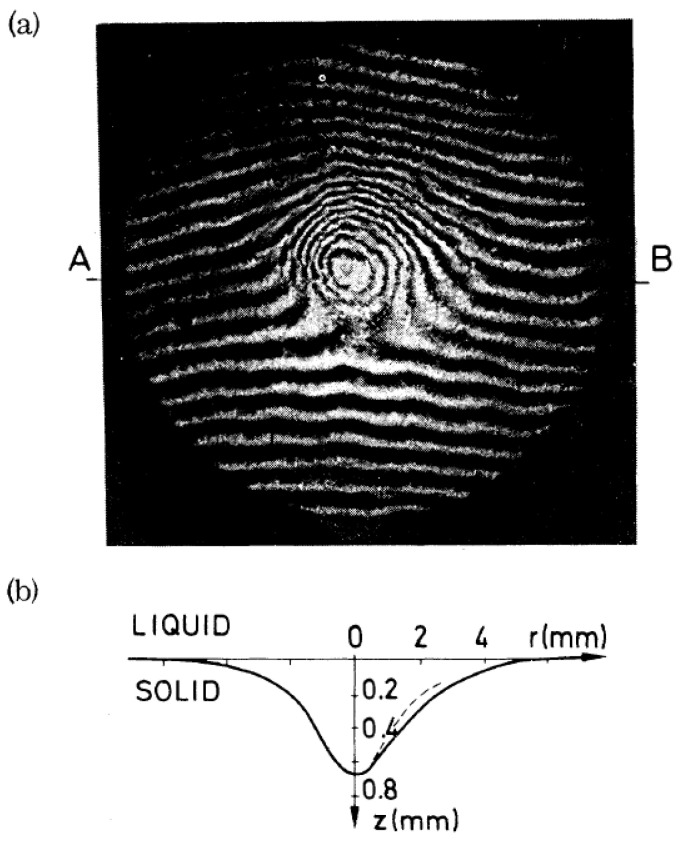
(**a**) Interference pattern of an hcp-superfluid ^4^He interface deformed by ∼10^6^ negative ions in an external field, *E* = 700 V/cm, at a temperature of *T* = 1.35 K. The parallel fringe pattern outside the center results from a slight inclination of the two interferometer plates. The helium crystal in this region is flat and, hence, the fringes are not distorted. (**b**) Dimple profile determined from (**a**) along the direction A–B (full curve). A comparison with the dashed line, which is a mirror image of the left-hand profile, illustrates the asymmetry of the dimple. Reproduced with permission from [32].

An experimental observation of the predictions of Equation (5) in the unstable regime is difficult with electrons at the liquid–vapor interface and also with ions at the interface of liquid ^3^He-^4^He mixtures, because—as already mentioned—the perturbation of the interface by the breakthrough of charges is chaotic, due to the fact that these surface waves are only weakly damped. However, the helium phases also provide yet another system, namely the interface between liquid and solid helium, which has already been introduced in Section 2. As mentioned there, the solid–liquid interface is found to trap negative ions from the liquid side. This is experimentally demonstrated in Figure 21, which shows the profile of a stable, negatively charged dimple at an hcp-superfluid ^4^He interface, measured interferometrically, as in Figure 20.

**Figure 22:**
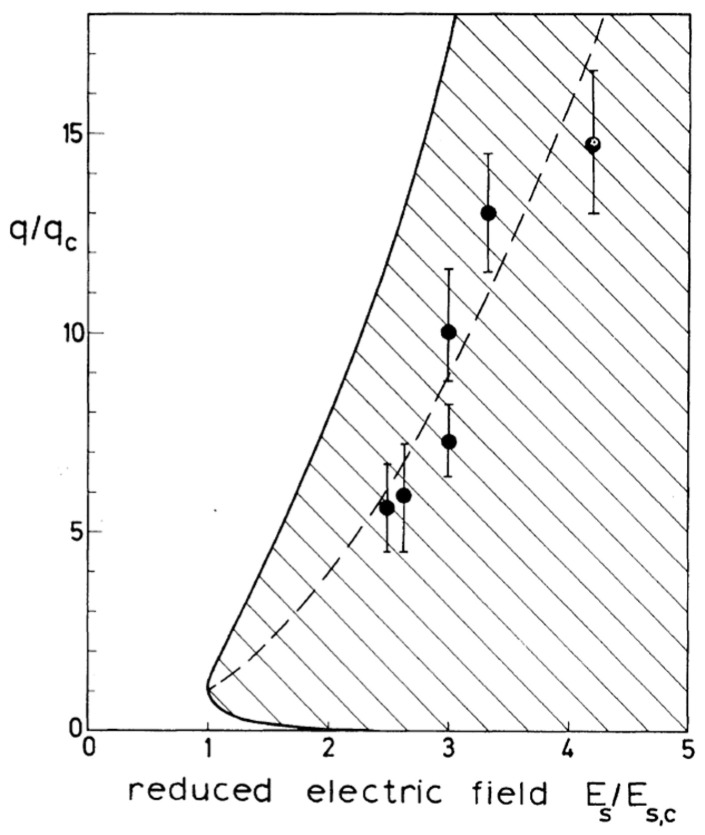
Calculated range of the charge-induced instability of the solid hcp-superfluid ^4^He interface, indicated by the hatched area, as calculated from Equation (1) in Ref. [32]. The solid line is a plot of the reduced wave vectors *q*/*q*_c_ for which the damping coefficient vanishes; the dashed line indicates the unstable wave vectors with the largest gain. The experimental results, given by the circles, were obtained by determining the characteristic average wave vector *q*_m_ of unstable, corrugated structures like in Figure 23. Reproduced with permission from [32].

This interface bears many similarities to a liquid surface, but in this case, the excitations are melting–crystallization waves instead of capillary waves [71,72]. The dispersion relation of these waves is similar to Equation (5) for a liquid–liquid or a liquid–vapor interface, with an additional term which takes into account the strong damping of the waves above 1 K due to the interaction with thermal rotons in the bulk liquid [32]. A stability diagram of this charged interface, derived from this dispersion equation, is shown in Figure 22, where the expected range of unstable wave vectors is plotted vs. the applied electric field, normalized by the critical field. It is expected that the band of unstable wave vectors becomes broader the higher the applied electric field, and that in particular, the dominating wave vector increases quickly with the quench depth.

**Figure 23:**
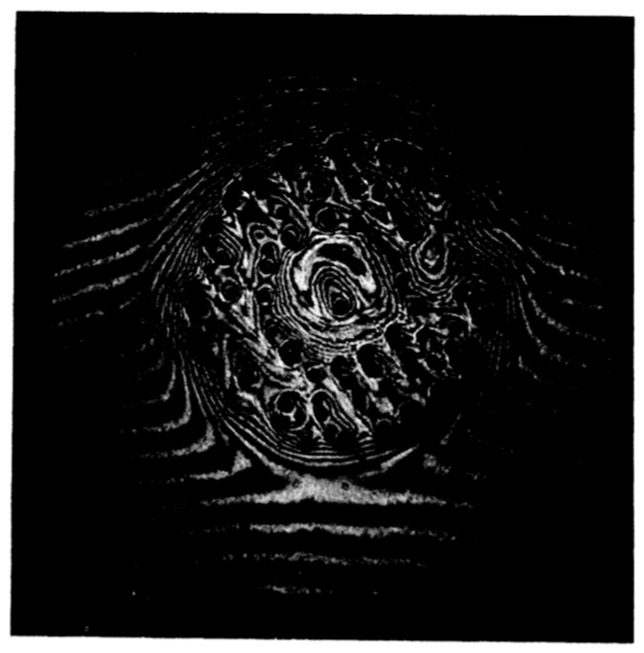
Interference pattern of an hcp-superfluid ^4^He interface undergoing a charge-induced instability, observed from above. The snapshot was taken about 1 sec after a (supercritical) voltage *U* = 1800 V had been applied between the top and bottom electrodes in the sample cell. An additional holding field resulting from negative charges on the cylindrical cell walls confines the ions on the crystal surface in a circular patch about 1 cm in diameter. Reproduced with permission from [32].

In the experiment [32], the charged liquid–solid interface was quenched into the unstable regime by a sudden rise in the electric field. A snapshot of the developing deformation of the interface is shown in Figure 23. Since the usual disturbances from waves reflected at the container walls are suppressed here due to their damping, the growth of unstable modes can be tracked over a timescale of seconds. Although the emerging pattern lacks the regularity of the dimple lattice seen in Figure 17, a degree of periodicity in the crystal’s deformation is still evident. This suggests that within the broad spectrum of unstable modes present at that field, a narrower subset dominates the dynamics. The orientation of the corrugations visible in Figure 23 reflects the anisotropic nature of the ^4^He crystal.

The deeper the quench, the faster the instability was found to develop. At the same time, the wave vector *q*_m_, which characterizes the spacing of the corrugations, grew larger but became less sharply defined. This led to a noticeable scatter in the data points shown in the stability diagram [Figure 22]. Even so, the correspondence with the theoretical curve for the fastest-growing modes—represented by the dashed line in Figure 22—is obvious, suggesting that the essential aspects of this instability have been captured.

### 4.4. Taylor Cone and Electrospraying

The final step of the EHD instability, a breakthrough of the charges at a helium interface, is the motion of charge packages to the counter electrode. In the case of surface-state electrons above the liquid, this breakthrough leads to multielectron bubbles [58,73], which have been studied in detail by Ghosh and coworkers [74,75]. In the opposite direction, the EHD instability of charges trapped *below* the liquid surface develops in the form of jets, or “geysers”. The first indirect hint towards the formation of such jets has been found in the previously mentioned work by Boyle and Dahm (see Section 2); their measurement of the discharge current from a liquid ^4^He surface charged from below with positive ions inferred the extraction of charged droplets (Figure 3) [24]. Volodin and Khaikin, in addition to an electrical measurement, took movies of the unstable superfluid helium surface charged from below by positive helium ions. They showed that the charges leave the helium surface, along with jets of liquid helium ejected by the ‘’geysers’’ arising on the crests of capillary waves [76].

**Figure 24:**
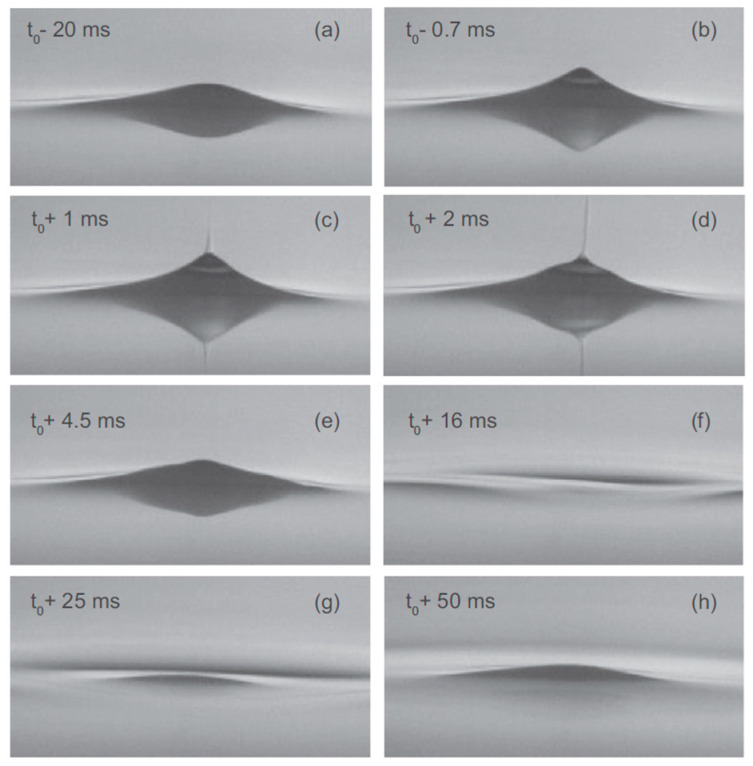
Frames of a fast video recording capturing the process of the charge escape from a Taylor cone at the surface of superfluid ^4^He. In this case, the top electrode was a pin ∼3 mm above the liquid surface; therefore, the charges are concentrated in the center of the sample cell. *U*_pin_ = −390 V, *U*_plate_ ≈ +900 V (ramp from +800 to +1800 V), *T* = 2.1 K, single frame exposure time ≈ 0.19 ms; time *t* = *t*_0_ corresponds to the beginning of the jet emission. The times *t* when the frames were taken with respect to *t*_0_ are indicated in the images (**a**–**h**). Frame size, 4.6 × 2.5 mm. Reproduced with permission from [77].

This phenomenon was studied in more detail by Moroshkin et al. [77], who, instead of the usual positive He ions (“snowballs”), used charged metallic micro- and nanoparticles, generated by laser ablation, to charge the surface from below. Also, in this case, cone-like structures developed—named Taylor cones [78]—which became unstable above a critical electric field, and a charged liquid helium jet was emitted. Figure 24 illustrates an example of the discharge process through a sequence of frames captured with a high-speed camera. At first, the cone exhibits a rounded apex (Figure 24a). As the breakthrough approaches, a sharp cusp forms at the tip (Figure 24b). From this cusp, a narrow jet of liquid helium is expelled upward (Figure 24c,d). The jet persists for roughly 3 ms and has a diameter of about 20–30 µm. Following the release of charge, the liquid surface beneath the tip drops below the level of the undisturbed fluid (Figure 24e,f). This sudden depression generates a circular wave that propagates outward from the center and dissipates within 50–100 ms (Figure 24f,g). Concurrently, a smaller residual cone forms at the center (Figure 24g,h). Just before the breakthrough, the cone’s apex angle *θ* approaches 100°, which closely matches Taylor’s theoretical prediction of *θ* = 98.6° [78].

## 5. Conclusions

Positive and negative ions at helium interfaces have been used in a broad spectrum of studies, including liquid–vapor, liquid–liquid and liquid–solid interfaces. Investigations of the trapping time of the ions at these interfaces have shown that qualitatively, the ions behave as expected; the details, however, are not accounted for by the existing theories based on the simple models for the negative electron bubbles and the positive snowballs. More realistic treatments have reduced the discrepancy between the calculated and the measured trapping times, and a “semiquantitative” agreement has been achieved [23]. More work will be necessary to come to a complete understanding of the ion transport across these interfaces.

Under experimental conditions where the interface provides a sufficiently high energy barrier, the ions can be trapped there for long times and form 2D Coulomb systems. This has been realized for positive and negative ions below the liquid–vapor interface of both pure ^4^He and ^3^He; at the interface of phase-separated ^3^He-^4^He mixtures, where negative ions have been kept above and positive ions below the interface, respectively; and at the liquid–solid interface with pools of negative ions from the liquid side. Some of these 2D Coulomb systems exhibit collective (plasmonic) excitations, which have been used to determine the characteristic parameters of the ions, like their mass and radius, but also to learn about the behavior of 2D systems in general, like the melting of 2D crystals. Furthermore, these trapped ions have been used as a sensitive probe for studying surface and bulk properties of helium—recently, in particular, the phases of superfluid ^3^He [51,52,53]—and more results of these intriguing systems are to be expected.

Finally, ions at helium interfaces have also been used as a model system for the EHD instability of charged liquid surfaces. All stages of this effect have been observed, starting from the softening of interfacial waves as a precursor of the instability—ions at the interface of ^3^He-^4^He mixtures are the only experimental system in which this softening has been measured so far. The broken symmetry of a new phase which appears at the critical point and the development of unstable modes in the regime beyond the instability complete the picture. EHD instability can thus also serve as a textbook example of this class of critical phenomena.

## Data Availability

No new data were created in this review.
